# Similarities between obesity in pets and children: the addiction model

**DOI:** 10.1017/S0007114516002774

**Published:** 2016-07-29

**Authors:** Robert A. Pretlow, Ronald J. Corbee

**Affiliations:** 1 eHealth International, 2800 Elliott Avenue #1430, Seattle, WA 98121, USA; 2 Department of Clinical Sciences of Companion Animals, Faculty of Veterinary Medicine, Utrecht University, Yalelaan 108, 3584 CM Utrecht, The Netherlands

**Keywords:** Childhood, Dogs, Cats, Co-dependence, Parental behaviour, Addiction, Obesity

## Abstract

Obesity in pets is a frustrating, major health problem. Obesity in human children is similar. Prevailing theories accounting for the rising obesity rates – for example, poor nutrition and sedentary activity – are being challenged. Obesity interventions in both pets and children have produced modest short-term but poor long-term results. New strategies are needed. A novel theory posits that obesity in pets and children is due to ‘treats’ and excessive meal amounts given by the ‘pet–parent’ and child–parent to obtain affection from the pet/child, which enables ‘eating addiction’ in the pet/child and results in parental ‘co-dependence’. Pet–parents and child–parents may even become hostage to the treats/food to avoid the ire of the pet/child. Eating addiction in the pet/child also may be brought about by emotional factors such as stress, independent of parental co-dependence. An applicable treatment for child obesity has been trialled using classic addiction withdrawal/abstinence techniques, as well as behavioural addiction methods, with significant results. Both the child and the parent progress through withdrawal from specific ‘problem foods’, next from snacking (non-specific foods) and finally from excessive portions at meals (gradual reductions). This approach should adapt well for pets and pet–parents. Pet obesity is more ‘pure’ than child obesity, in that contributing factors and treatment points are essentially under the control of the pet–parent. Pet obesity might thus serve as an ideal test bed for the treatment and prevention of child obesity, with focus primarily on parental behaviours. Sharing information between the fields of pet and child obesity would be mutually beneficial.

Obesity in companion animals is an important disease with a prevalence in dogs of 33–60 %^(^ ^1^ ^–^ ^4^ ^)^. In cats, the prevalence of obesity is reported to be 11·5–27 %^(^ ^5^ ^–^ ^7^ ^)^, although a more recent article on obesity in show cats revealed a prevalence of 45·5 %^(^ ^8^ ^)^. Equine obesity prevalence is reported to be 27–54 %^(^ ^9^ ^–^ ^12^ ^)^.

Similarly, in human children, the prevalence of combined overweight and obesity, worldwide, rose by 47·1 % between 1980 and 2013^(^ ^13^ ^)^. In 2013, 23·2 % of children were overweight or obese in developed countries and 13·2 % in developing countries^(^ ^14^ ^)^.

As obesity in pets is related to several other diseases (i.e. osteoarthritis, osteochondrosis, laminitis, CVD, hepatic lipidosis and diabetes mellitus^(^ ^15^ ^–^ ^18^ ^)^ as well as decreases life span^(^ ^19^ ^)^), it is imperative to investigate the causes of obesity and to provide guidelines for treatment and prevention. Akin to pets, diseases associated with obesity in children (e.g. type 2 diabetes, non-alcoholic fatty liver disease, dyslipidaemia, hypertension, polycystic ovarian syndrome and sleep apnoea) are surging^(^ ^20^ ^)^.

## Interventions

Obesity in pets and children is a frustrating, intractable problem. Interventions in children have produced modest weight loss at short term, but the weight is often regained in the long term^(^ ^21^ ^,^ ^22^ ^)^. In dogs, only half of 61 % that initiate a weight-loss regimen successfully complete it^(^ ^23^ ^,^ ^24^ ^)^, and approximately half of the dogs that successfully reach goal weight subsequently regain weight^(^ ^18^ ^)^. Obese pet dogs that successfully lose weight and maintain the weight are the minority. The author speculates that ‘Obesity in pets may be an intriguing model for childhood obesity’^(^ ^25^ ^)^.

## Similarity of causality

Poor nutrition and sedentary activity are intuitive contributors to the rising obesity rates in both children and pets. However, what is intuitive may not actually be true. Indeed, increase in fat mass has been found to cause sedentary activity rather than the reverse^(^ ^26^ ^)^. Other explanations for the causes of the child obesity epidemic include genetics (thrifty gene), weight set-point, low metabolism, food-rich environment, cost of healthy, low-energy food and poor food choices. These explanations may also apply to pets; nevertheless, none of these explanations has been established.

A novel explanation for child obesity stems from the observation that parents give ‘treats’ to their children and extra food at meals to gain affection and love from the children^(^ ^27^ ^)^. Food companies understand this – for example, the Cool Whip Commercial ‘Give the Cool Whip, Get the Love’ http://www.weigh2rock.com/videos/food_for_love.html. A parent may become psychologically dependent (addicted) to this treat-induced affection, and in the process enable eating addiction in the child, as a means of positive interactions with the parent^(^ ^28^ ^)^.

The term for this is ‘co-dependence’. Both the parent and the child become psychologically dependent on the treats and extra food given to the child. Actual addictive tolerance may develop, such that the child demands more and more treats/food and higher pleasure-level foods^(^ ^29^ ^)^, or the parent independently may insist on more treats/food for the child. Several parents in our recent study argued that their children were not getting enough when reducing food amounts, even though the children were not losing weight or were continuing to gain weight^(^ ^30^ ^)^. Grandparents and other close family members (aunt, uncle, etc.) also may be a part of this co-dependence enabling overeating^(^ ^31^ ^)^.

At some point, the parent realises that the child has become overweight and may try to cut back on the treats and extra food. Parental withdrawal symptoms, resulting from loss of affection from the child, thanks to fewer treats, may prevent the parent from doing so^(^ ^28^ ^)^. A 270-pound, 13-year-old boy in our recent study was attempting to cut down on lunch amounts at school by personally packing his lunch. His mother insisted on adding candy ‘treats’ in his lunch. When she was asked not to do so, she replied, ‘It’s my thing’. She discontinued from the study^(^ ^30^ ^)^.

The child, likewise, does not want to go through withdrawal and may shun the parent or become overtly angry because of loss of ‘food fixes’. Aversion to ‘cold shoulder’ or anger from the addicted child then perpetuates the parental enabling. The mother of a 287-pound, 10-year-old boy in our study reported that she gave treats to her son, because he would get very angry if she did not^(^ ^30^ ^)^.

This probably contributes to the fact that half of companion animals currently are overweight or obese. Pets are like children to many pet owners^(^ ^32^ ^)^. Similar to human parents, ‘pet–parents’ give treats/extra food to their pets to garner perceived love and positive interactions from their pets^(^ ^33^ ^)^, which may result in the pets becoming addicted to the foods. Pet–parents, as well as other members of the ‘pet-family’, likewise may develop co-dependence on the treats/food as a result of the affection garnered from the pet. Pet food commercials tend to portray this: ‘Feed the dog good food and get the real love’ by Iams (https://www.youtube.com/watch?v=NG0MKXxdhrw). Furthermore, dogs easily learn how to get extra food, as well as learn how to respond if they do not get the extra food, thus demonstrating the same evasion of withdrawal symptoms as humans^(^ ^34^ ^–^ ^36^ ^)^.

Eventually, the pet–parent realises that the pet is becoming overweight and attempts to curb the feeding. Both the pet–parent and the pet then experience withdrawal symptoms, and the pet may become unaffectionate as a result^(^ ^37^ ^)^. As in human relationships, the pet–parent’s fear of rejection may surpass the desire for love. Rejection hurts! The pet may even become aggressive or dominant and bite or show negative behaviours towards the pet–parent or others in the household when food is restricted (RJ Corbee, unpublished results). Thus, the pet–parent is held ‘hostage’ to the extra food and treats to keep the pet happy.

## Stress eating

It is well known that human behaviours such as nail biting and skin picking are associated with stress or anxiety. The brain tends to readily adopt (hijack?) any behaviour that eases stress or anxiety, which may progress to a behavioural addiction. Stress eating or ‘nervous eating’ is an example. In *Stress in America* ^(^ ^38^ ^)^, findings suggest substantial stress levels in children and a relationship between their stress and obesity. Children who are overweight are more likely to report that they worry a great deal about things in their lives than children who are normal weight (31 *v*. 14 %). Children who are overweight are more likely than children of normal weight to report eating to make themselves feel better when they are really worried or stressed about something (27 *v*. 14 %).

Stress in animals can cause overeating and obesity. In the 1970s and 1980s, extensive research was conducted on stress-induced eating in laboratory rats. In one study entitled ‘Stress-induced hyperphagia and obesity in rats: a possible model for understanding human obesity’, mildly stressing a rat by placing a padded clamp on the rat’s tail very reliably induced overeating of standard rat chow and resulted in weight gain in multiple sated animals. This progressed to the point of obesity^(^ ^39^ ^)^. Exposing the rat to annoying noise likewise induced overeating of standard rat chow. Stress-induced eating in the rat may represent ‘displacement behaviour’^(^ ^40^ ^)^. Displacement behaviour is commonly observed in the wild in most animals as a response to stress. This behaviour is characterised by actions typically inappropriate for the situation but which bring comfort to the animal, such as licking or feeding when threatened by a predator^(^ ^41^ ^)^. Stressed dogs may lick their paws to the point of injury (‘lick granuloma’)^(^ ^42^ ^)^, and cats similarly lick their lips when threatened. McMillan^(^ ^43^ ^)^ reviewed evidence for stress-induced eating in companion animals and stated that ‘excessive eating and obesity may be a result of a diminished quality of life’. For example, cats kept in apartments have a higher prevalence of obesity than cats with outside access. Limited space may not only induce obesity in pets by lack of exercise (lack of energy expenditure) but also by distress-induced eating caused by a lack of environmental enrichment and being unable to express natural behaviour^(^ ^44^ ^)^. Even though pets have limited access to food compared with children, pets may comfort/stress eat by begging when in distress, similar to children pestering parents if food access is restricted^(^ ^43^ ^)^.

## Eating addiction

Initially, children and pets may overeat because ‘the food is there’ – it simply tastes good or is fun. Nevertheless, if depression, isolation, anxiety, stress or boredom is eased by the pleasure or action of eating, this ‘comfort eating’ behaviour will be repeated, typically mindlessly. As the children and pets continue to eat to ease psychological distress, changes take place in their brains to reinforce the behaviour and keep it going^(^ ^45^ ^)^. When significant brain changes have taken place, the children and pets are unable to stop comfort eating, and it becomes an addictive process^(^ ^46^ ^,^ ^47^ ^)^.

Accordingly, the combination of circumstances present in the Western world may be contributing to the childhood obesity epidemic: (a) cheap, widely available, highly palatable foods, (b) increased distress in children and (c) comfort/stress eating, leading to eating addiction. Similar circumstances may correspondingly exist with pets: (a) highly palatable foods are easily available and are being fed *ad libitum*, (b) lack of environmental enrichment, probably resulting in increased distress in pets and (c) comfort/stress eating (also initiated by pet owners who feed treats to calm down the pet in stressful situations), leading to eating addiction when pets experience chronic distress^(^ ^43^ ^)^.

Actual tolerance may also develop: a 5′2″, 201-pound, 14-year-old girl exclaimed ‘It’s like a drug. What used to satisfy you before now has no effect. I feel like I’ve become immune to the foods that used to comfort me. And like drugs you keep moving on to bigger, worse things in order to get the same feeling as when you started out’^(^ ^46^ ^)^. Thus, kids will eat larger amounts and higher pleasure-level foods to obtain the same degree of comfort. Pets may follow a similar pattern; they will increase their begging behaviour (especially when it was proven to be effective before), which results in getting more and more treats and food from the owners^(^ ^34^ ^,^ ^36^ ^,^ ^48^ ^)^.

A real-life example is a moving story of a morbidly obese child/adult who sought comfort in food and whose mother said that he would become angry if she did not give him the food he wanted (http://www.huffingtonpost.com/2015/01/17/hectorgarciaobesity-photos_n_6492354.html). As the mother admitted, ‘they were the wrong things, but I would give in because I was so tired’. Nobody likes to be yelled at or cold shouldered, especially over something as silly as food. There are similar long-term stories among pet owners, as was demonstrated in a study by Jakovcevic *et al*.^(^ ^36^ ^)^. Another example is the fact that owners of obese cats in weight-loss programmes mentioned about their cats waking them in the middle of the night to be fed, which is a major problem for compliance in feline weight-loss programmes (RJ Corbee, unpublished results).

An obese parent may simply overeat along with the child, and the child thereby acquires the parent’s eating addiction. A mother in our parents’ chat room confessed, ‘Sometimes when me and my daughter sit down with piles of junk food and 4 hours later I look around and the amount of food and calories we have eaten amazes me. It is like a drug for her and I’^(^ ^46^ ^)^.

Pet–parents may likewise be obese^(^ ^49^ ^)^. Obese people commonly fill their homes with junk food and meals consist of prolific portions. When pets see the pet–parent constantly eating, the pet may develop the habit of begging^(^ ^34^ ^)^.

## Similarity of treatment: the addiction model

Traditional treatment of weight gain in both children and pets involves ‘lifestyle modification’ of diet and exercise. Such approaches have failed to produce significant long-term weight loss in children^(^ ^50^ ^)^ or in pets^(^ ^25^ ^)^. Mounting evidence suggests that overeating/obesity in children involves an addictive process^(^ ^46^ ^,^ ^51^ ^–^ ^53^ ^)^.

Accordingly, Pretlow *et al*.^(^ ^30^ ^)^ have developed an obesity intervention for children based on classic addiction medicine techniques of withdrawal/abstinence, combined with behavioural addiction treatment methods. In this intervention, children and parents go through a procedure of staged food withdrawal. First, withdrawal takes place from ‘problem foods’, which are specific foods characterised by cravings and difficulty resisting the food when immediately available. The children withdraw from each problem food, one-by-one, by total abstinence from the food for a minimum of 10 d in a row. Parents, likewise, go through withdrawal by not having the problem foods in the home or offering such treats outside the home. Problem food withdrawal is followed by withdrawal from eating between meals (snacking – non-specific foods). Finally, reduction in portion amounts at meals is tackled by incrementally reducing starting portion amounts until weight loss occurs. Further reductions are titrated against the desired amount of ongoing weight loss. Behavioural addiction treatment methods in the intervention include alternative behaviours (e.g. walking, squeezing hands), lessening of stress, distractions and distress tolerance (e.g. ‘urge surfing’). Trigger avoidance such as staying out of the kitchen and having no food in sight except specific portions at meals enhances this approach. A pilot trial in children showed significantly positive results^(^ ^30^ ^)^.

The approach of staged food withdrawal, together with behavioural addiction treatment methods, should adapt well for treatment of obesity in pets, despite the fact that their metabolism is different compared with humans. Both the pet and pet–parent would go through gradual withdrawal from treats and excessive amounts at meals. The pet–parent would need to implement ‘tough love’ and tolerate ‘cold shoulder’ and actual hostility from the pet when reducing treats/food, as well as seek alternative sources of companionship. If a specific treat/food can be totally abstained from or amounts reduced to a new level for at least 10 d in a row, the pet’s behaviour will change, and cravings and begging should cease^(^ ^36^ ^)^. Keeping food out of sight helps tremendously to quell begging. The pet–parent may need to confront and be treated for her/his own addictive eating to cease enabling it in the pet.

Behavioural addiction methods successfully used in obese children and their parents that can be adapted for pets and pet–parents include the following: (a) avoiding triggers (e.g. staying out of the kitchen, combating boredom), (b) distractions (e.g. walking or playing with the animal), (c) distress tolerance (e.g. tolerating begging – feeling the urge to give food to the pet but risking not acting on it), (d) taking a deep breath, holding it for a few seconds, and then letting it out and (e) squeezing the hands together. Decreasing portion amounts for the pet could be facilitated by using smaller scoops and smaller feeding bowls^(^ ^54^ ^)^, keeping the pet away from extra food (e.g. serving food in one room and eating it in another) and keeping the pet away from humans eating food. Uneaten food should immediately be removed from the pet’s dish. An additional problem in pet obesity lies in multiperson households. As pets cannot speak, they will constantly take treats from every person in the household. As all caregivers like to give food and treats to their pets, the behavioural addiction methods should be used by all caregivers.

Taking the pet to ‘pet day care’ while at work or school would avoid stress eating due to isolation. Fun activities such as taking the pet outside, walking the animal, visiting a park or friends with similar pets could avert boredom at other times^(^ ^55^ ^)^.

The habits of dog owners to give treats after walks or outings with the owner should also be controlled, as well as the use of treats for training purposes.

## De-pleasurising mealtime foods

De-pleasurising mealtime foods is a further form of withdrawal to accomplish portion reduction. If foods are not as tasty, children and pets tend to eat less. For example, in children, if there is a particular problem with mashed potatoes, which typically are made with butter, cream and seasoning, they can be prepared as boiled potatoes with just seasoning. Increasing the fibre content and decreasing the fat content of pet food are ways to reduce energy consumption^(^ ^16^ ^)^. Pets and children may be annoyed by this de-pleasurising and initially refuse the less tasty foods. Perseverance by the pet–parent and child–parent is key, if this theory holds true for pet eating behaviours.

## Withdrawal symptoms

The study of Pretlow *et al*.^(^ ^30^ ^)^ found that, with a few notable exceptions, withdrawal from problem foods and snacking in children was associated with minimal withdrawal symptoms. However, withdrawal from excessive food amounts at meals was associated with significant withdrawal symptoms including nagging urges, agitation and even anger. Hence, in pets, withdrawal from excessive amounts at meals may prove more difficult than withdrawal/abstinence from specific treats.

The high rate of relapse of obesity in both pets and children suggests significant withdrawal symptoms in co-dependent pet–parents and child–parents. In the Pretlow *et al*.^(^ ^30^ ^)^ study, many parents felt their child was not ‘getting enough’ in spite of negligible weight loss or even weight gain. Pet–parents, similarly, feel compelled to keep adding more food to the pet’s dish. For pets, 2 discrete meals/d, with no food or treats in between, is the goal. Actually weighing out portions with a food scale is the optimal method by which to incrementally reduce amounts, as it avoids the withdrawal indecision of how much to add to or remove from the dish. A 17-year-old girl in our study commented, ‘It works because it’s an “exact number,” I can’t add more or less, there’s no decision’. A 10-year-old girl stated ‘If you eyeball it, it lets you get away with more than you’re actually supposed to have’^(^ ^30^ ^)^. Indecision anxiety may result in cheating by the pet–parent or child–parent, as anxiety tends to be dealt with by providing food for the pet/child to get love – or giving in to begging. Withdrawal symptoms may recur after each portion reduction increment; nevertheless, such symptoms typically abate 2–3 d after each reduction. Weighing out and freezing several meals at once is a way to further minimise indecision anxiety of how much to dish out or put back.

## Discussion

Initially, pet–parents and child–parents give treats/extra food to gain affection/love received in response (positive reinforcement). Once treats are given to avoid a cold shoulder or hostility from the pet/child, the co-dependence transitions into negative reinforcement (avoiding emotional pain) on the part of the pet–parent or child–parent. Both the pet–parent and child–parent then become hostage to the treats/food.

Staged food withdrawal from treats/extra food is effective in obese children and their parents, and should be relatively easy to implement in obese pets and pet–parents. Behavioural addiction methods also should be easily adapted for pets and pet–parents, such as avoiding triggers (kitchen, boredom), distractions (walking, playing) and distress tolerance (urge surfing). Decreasing portions for the pet should be possible with smaller scoops and smaller feeding bowls, keeping the pet away from extra food, as well as keeping the pet away from humans eating. Weighing out portions, which is effective in children, is a further option for pet–parents.

Pet obesity is more of a caregiver-dependent obesity than child obesity, in that contributing factors and treatment points are essentially under the control of the pet–parent. In contrast, human children may obtain food independently at school, the corner store or a friend’s or relative’s house. Pets generally are limited to food in the pet–parent’s house, even though the pet too may eat a neighbour pet’s food (e.g. enter through the cat door) or be offered food/treats by a neighbour. Thus, staged food withdrawal could be more feasible in pets.

Pet–parent co-dependent difficulty in controlling feeding the pet may shed light on child–parent co-dependent enabling of eating addiction in children. Moreover, pet obesity may serve as an ideal test bed for caregiver-dependent obesity in investigating causes, treatment and prevention of child obesity.

Stress eating in pets is not enabled by co-dependence of pet–parents, but caused rather by emotional and/or social negligence by the pet–parent. Measures to avoid such neglect are fairly straightforward. In extreme cases, an organisation such as a human society may need to intervene.

### Conclusions

The causes of pet and child obesity are strikingly similar. Accordingly, treatment should basically follow the same approach. Staged food withdrawal, along with behavioural addiction treatment methods, is appropriate for both overweight children and overweight pets. Ostensibly, this approach should be more feasible in pets, as access to food is more controllable in pets. Nonetheless, pet–parent co-dependence is likely as daunting to treat as child–parent co-dependence. Information garnered from each field should complement treatment and prevention in the other.
